# Computational tools for rational protein engineering of aldolases

**DOI:** 10.5936/csbj.201209016

**Published:** 2012-11-13

**Authors:** Michael Widmann, Jürgen Pleiss, Anne K. Samland

**Affiliations:** a Institute of Technical Biochemistry, University of Stuttgart, Allmandring 31, 70569 Stuttgart, Germany; b Institute of Microbiology, University of Stuttgart, Allmandring 31, 70569 Stuttgart, Germany

**Keywords:** protein engineering, substrate specificity, stereoselectivity, *de novo* design, transaldolase, biocatalysis

## Abstract

In this mini-review we describe the different strategies for rational protein engineering and summarize the computational tools available. Computational tools can either be used to design focused libraries, to predict sequence-function relationships or for structure-based molecular modelling. This also includes *de novo* design of enzymes. Examples for protein engineering of aldolases and transaldolases are given in the second part of the mini-review.

## 1. Introduction

Asymmetric aldol additions are a corner stone of preparative organic chemistry. Concomitant with the formation of a C-C bond between a nucleophile (donor) and an electrophile (acceptor) one or two new stereocenters are created. This type of reaction can also be carried out by enzymes, such as aldolases and transaldolases. Those enzymes, in most cases, strictly control the stereo configuration at the newly formed stereocenter(s). Aldolases are applied in biocatalysis for the synthesis of amino acid and carbohydrate derivatives. For more details about aldolases and their biocatalytic application see recent reviews [1–4].

Mechanistically, class I and class II aldolases are distinguished. Class I aldolases form a Schiff base intermediate between a conserved Lys in the active site and the carbonyl carbon atom of the donor substrate, i.e. usually a ketone. By proton abstraction an enamine intermediate is formed which attacks the carbonyl carbon atom of the acceptor aldehyde. Class I aldolases do not require any cofactor and they exhibit a typical (β/α)_8_-barrel fold. Class II aldolases depend on a divalent cation which acts as a Lewis acid. The metal ion helps to deprotonate the donor substrate and stabilises the enolate formed. Therefore, these aldolases can be inhibited by EDTA. According to their structure and sequence class I and class II aldolases do not show any significant homology. Apparently, they evolved separately.

Aldolases usually accept a wide range of acceptor substrates which allows a broad range of synthetic applications. On the other hand, they are in general very specific for their donor substrate. Hence, they are classified as (i) dihydroxyacetone phosphate (DHAP) dependent aldolases, (ii) dihydroxyacetone (DHA) dependent aldolases, (iii) pyruvate/2-oxobutyrate dependent aldolases, (iv) acetaldehyde dependent aldolases and (v) glycine/alanine dependent aldolases [1]. Glycine/alanine dependent aldolases are neither class I nor class II aldolase but require pyridoxal phosphate (PLP) as cofactor. Structurally, they belong to the fold type I family of PLP dependent enzymes.

Transaldolases (Tal) transfer a DHA moiety from a ketose donor to an aldehyde acceptor. A new C-C bond is formed with 3*S*,4*R* stereo configuration. Mechanistically (Schiff base intermediate) and structurally ((β/α)_8_-barrel fold), Tals show similarity to class I aldolases. However, compared to DHAP dependent class I aldolases the conserved Lys residue moved to a different β-strand suggesting a circular permutation of the protein sequence [5]. Tals are almost ubiquitous enzymes and according to their sequence similarity they were divided into five subfamilies. The wild type enzyme did not find much application in biocatalysis. For more details on the Tal enzyme family see recent publications [6, 7].

Using computational tools protein engineering within this enzyme family was directed towards the following aims: (i) the discovery of new enzymes, (ii) the differentiation between enzyme families or subfamilies, (iii) the engineering of enzymes for new applications and (iv) the design of novel aldolases. In this mini-review we will first describe the different strategies for protein engineering and summarize the computational tools available. In the second part, we will give examples from the enzyme family of aldolases and transaldolases.

## 2. Computational tools for protein engineering

Isolated enzymes have been successfully applied for bioconversions provided the enzyme is stable, soluble, and easy to produce. However, in most cases the commercially available enzymes are not optimal for the desired chemical process. Therefore, *in silico*, *in vitro*, and *in vivo* strategies have been developed to screen for appropriate enzymes from the natural pool [8]. However, natural enzymes rarely have the combined properties necessary for industrial chemical production such as high activity, high selectivity, broad substrate specificity towards non-natural substrates, no inhibition by substrate or product, and a high stability in organic solvents and at high substrate or product concentrations [9]. Therefore, protein engineering has been successfully applied to design enzymes with new substrate spectra and new functions as catalysts for unnatural substrates, and to fine-tune bottleneck enzymes in metabolic engineering [10]. Three major computational strategies are currently applied to support protein engineering: directed evolution, methods to predict sequence-function relationships, and structure-based molecular modelling methods.

### 2.1 Design of focused libraries for directed evolution

Directed evolution has proven to be an effective method to improve the properties of enzymes (for aldolases see review [11]). The unguided use of random mutagenesis methods, however, results in protein libraries with millions of members which still only sample a small fraction of the vast sequence space possible [12]. Recently, several computational approaches have been suggested to improve the efficiency of the directed evolution by enriching the library and reducing the library size substantially, taking into account further information. An enrichment of the library may be achieved by considering structure information on residues that are involved in substrate binding. This approach has guided the design of highly focused libraries and resulted in mutants with increased selectivity [13–15] or shifted substrate specificity [16–19]. The size of the library can be reduced by limiting the possible amino acid alphabet, i.e. not all 20 amino acids but a subset is used instead, depending on the desired interactions [20]. To estimate the screening effort necessary the CASTER tool was developed by the Reetz group. A comprehensive statistical analysis of a large number of favourable and less favourable mutants identified hot spot regions that are beneficial to enzyme activity and stability [21–23]. Most of these methods to search for promising mutation sites require expert knowledge in bioinformatics which may not be present in experimentally oriented research groups. Therefore, online tools that require little to none bioinformatics knowledge have become popular. Meta-tools such as the HotSpot Wizard [24] offer a complete workflow to assess promising mutation sites by combining a variety of methods such as Catalytic Site Atlas [25], CASTp [26], CAVER [27], BLAST [28], MUSCLE [29], as well as sequence and structure databases such as UniProt [30], NCBI GenBank [31], and PDB [32].

### 2.2 Prediction of sequence-function relationships

The second strategy takes advantage of the rapidly growing amount of available protein sequences, structures, functional and biochemical data. Systematic analyses are based on large number of protein sequences and complete protein families to yield insights into catalytic mechanisms and evolutionary pathways [33]. By comparing the sequences of homologous proteins, consensus or ancestor sequences were constructed. Back-to-the-consensus mutations were shown to increase stability [34–36] or improve expression [37]. Recently, ancestral mutations have been integrated with directed evolution to generate a stabilized starting point of highly diverse and evolvable gene libraries [38]. Alternatively, multi-sequence alignments were analyzed to identify correlated mutations, to identify structurally or functionally relevant residues [39, 40], and to predict mutants with improved substrate specificity, catalytic activity, or protein stability [41]. Sequence-based methods were also applied to predict aggregation-prone regions [42] and to design mutants with decreased aggregation rates [43]. Multiple sequence alignments assisted by structural information were also used to identify subfamily specific positions in aldolases [44–46].

While the amount of information on sequence, structure, and biochemical information is steadily increasing, it is generally not available to a systematic analysis. Therefore, databases have been developed that provide access to enzymatic information such as BRENDA [47] or to integrate information on enzyme families such as DWARF [48] and 3DM [49]. BRENDA (BRaunschweig ENzyme DAtabase) offers a comprehensive collection of biochemical data on a broad range of enzyme families, which are grouped according to their EC numbers, providing information about reaction type, products, and substrates, organisms of origin, and an overview of available publications. The DWARF system (Data Warehouse system for Analyzing pRotein Families) integrates sequence, structure, and annotation information of large protein families including lipases [50], triterpene cyclases [51], thiamine-diphosphate dependent enzymes [52], and lactamases [53]. The 3DM system [54] is based on the creation of structure-based multiple sequence alignments. A common numbering scheme for structurally equivalent amino acids allows for the automated creation of homology models, the analysis of correlated or conserved residues and the prediction of functionally relevant residues [41, 55]. As of the time of this review, no database with a focus on aldolases has been published.

### 2.3 Structure-based molecular modelling

The third strategy starts from information on protein structure and seeks to improve stability, activity, specificity, or selectivity by molecular modelling. While for a growing number of proteins, experimentally determined structure information become available by the Protein Data Bank [32], only for a small fraction of all proteins with known sequence the structure is also known. However, if sequence similarity is sufficiently high the structure of a protein can be modeled based on a sequence comparison to a protein with experimentally determined structure. Sequence identities as low as 25% are usually enough to predict reliable structure models, in some cases even sequences with lower sequence identities are suitable for homology modeling [56]. Homology modeling programs such as Swiss-Model [57], Modeller [58] or Rosetta [59] are based on the observation that during evolution structure has been more conserved than sequence. Thus, proteins with similar sequence have a similar structure. Using these methods, structure models can be derived for the majority of soluble proteins as demonstrated by the biannual Critical Assessment of Protein Structure Prediction [60].

Many strategies for protein stabilization have been proposed: optimization of the distribution of surface charge–charge interactions [61, 62], improvement of core packing [63] and of the protein surface [64], and rigidification by introduction of prolines, exchange of glycines, introduction of disulfide bridges [65] or mutagenesis at positions with high B-factor [66]. However, it is still challenging to reliably predict mutations that stabilize the enzyme without affecting its activity or selectivity, which are a direct consequence of the molecular recognition of the substrate by the enzyme. For a change in stereoselectivity the side chains in vicinity of the stereocentre can be determined from structural data. These residues can then be split into sectors containing two to three residues which are randomized simultaneously [67, 68]. To improve activity and selectivity, modelling of the enzyme-substrate complex by molecular docking methods has been used to study the molecular basis of specificity and selectivity, and to predict mutations in the enzyme or modifications of the substrate structure that mediate specificity or selectivity [69–71]. It is recognized that shape and physico-chemical properties of the active site and the substrate binding site are the major driving forces to provide the specific interactions between enzyme and the transition state of the substrate that lead to catalysis. Moreover, there is increasing evidence that flexibility of the enzyme-substrate complex is crucial to recognition, because minor structural adjustments can have a big impact on the docking score [51]. Docking has been extensively used to predict substrate specificity and to identify positions that mediate substrate binding. Amino acids that clash with the desired substrate upon docking were exchanged, leading to an increase of catalytic activity of the enzyme variant toward this substrate [72–74]. Catalytic activity is mediated by only a small number of amino acids, metals, or cofactors located in the vicinity of the active site. However, substrate specificity and selectivity of an enzyme might be determined by factors beyond the geometric shape of the active site, such as long-range effects of mutations [75, 76] or the effect of a substrate access tunnel [77, 78]. Methods of simulating protein structure and dynamics have been successfully applied to investigate the molecular basis of thermostability [79], temperature optimum [80], or specificity and selectivity [81–86]. Simulations are already successfully applied as powerful tools to interpret experimental results in retrospect, and we are only at the beginning of applying these methods for a predictive, rational design of enzymes.

Though fine-tuning enzymes by point mutations has been successfully applied, the resulting enzymes are still limited to a small range of reactions catalysed by natural enzymes. Therefore, a major challenge of enzyme design is to go beyond the range of natural reactions and to design enzymes with new catalytic functions. The strategy of transplanting a chemical activity takes advantage of enzyme promiscuity [87, 88] and has been successfully applied to engineering a lipase into an aldolase [89]. Beyond this, first successful steps have been made towards *de novo* design of enzymes that have a new catalytic function: a retro-aldol enzyme was designed which showed a rate acceleration of the catalysed versus the uncatalysed reaction by 10^4^ [90]. However, while the *de novo* designed enzymes are functional, their catalytic efficiency is still many orders of magnitude below the efficiency of natural enzymes [91]. The efficiency of *de novo* designed enzymes can be increased by directed evolution (see section 3.4 ) [85].

## 3. Selected examples for engineering of aldolases and Tals

We will demonstrate on a few examples how computational tools were used successfully for engineering of aldolases and transaldolases. This includes engineering approaches using localised randomisation, i.e. saturation mutagenesis, at positions which have been predicted to be important for the engineered property based on structural information or models. Unguided use of random mutagenesis, e.g. error prone PCR (epPCR) or DNA shuffling, is beyond the scope of this review. For more examples, also of evolutionary approaches, see recent reviews [2, 11].

### 3.1 Alteration of substrate specificity

**Figure 1:**
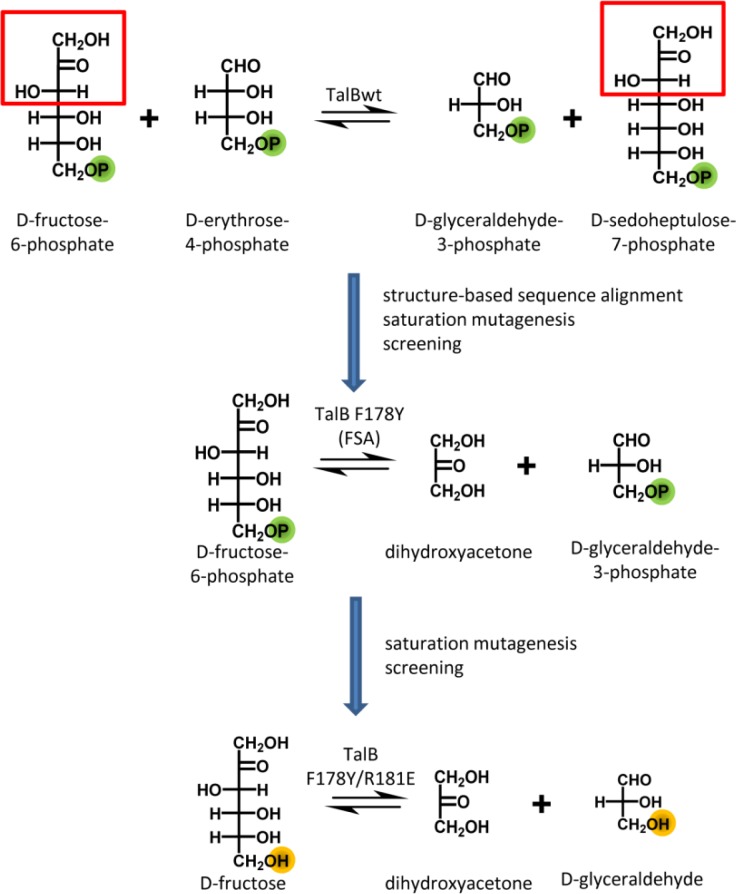
**Protein engineering of TalB**. First, TalB was engineered to use DHA as donor in an aldolase reaction. In a second step, the affinity towards non-phosphorylated acceptors was improved.

The high affinity or strict specificity of aldolases towards phosphorylated substrates is a major limitation in their biocatalytic application. Phosphorylated substrates are often instable and expensive and the phosphoryl group introduced in the product needs to be removed from the final product. Therefore, aldolases with higher affinity for non-phosphorylated acceptor and donor substrates are highly desired. The binding site of the phosphoryl group of the acceptor substrate in a recently engineered DHA dependent aldolase (TalB F178Y)[46] was identified due to a sulphate ion which was bound in the active site in the crystal structure [46]. The coordinating positions (2x Arg, 1x Ser) were targeted by saturation mutagenesis. The generated mutant libraries were screened using a newly developed colour assay for variants exhibiting a higher affinity for the non-phosphorylated acceptor D-glyceraldehyde [18]. Positive clones were identified in the library at position TalB F178Y/R181X. The best results were achieved for the TalB F178Y/R181E variant with an at least 2-fold improvement in affinity for D-glyceraldehyde (Fig. 1). This confirmed the importance of R181 for binding of the phosphoryl group.

**Figure 2:**
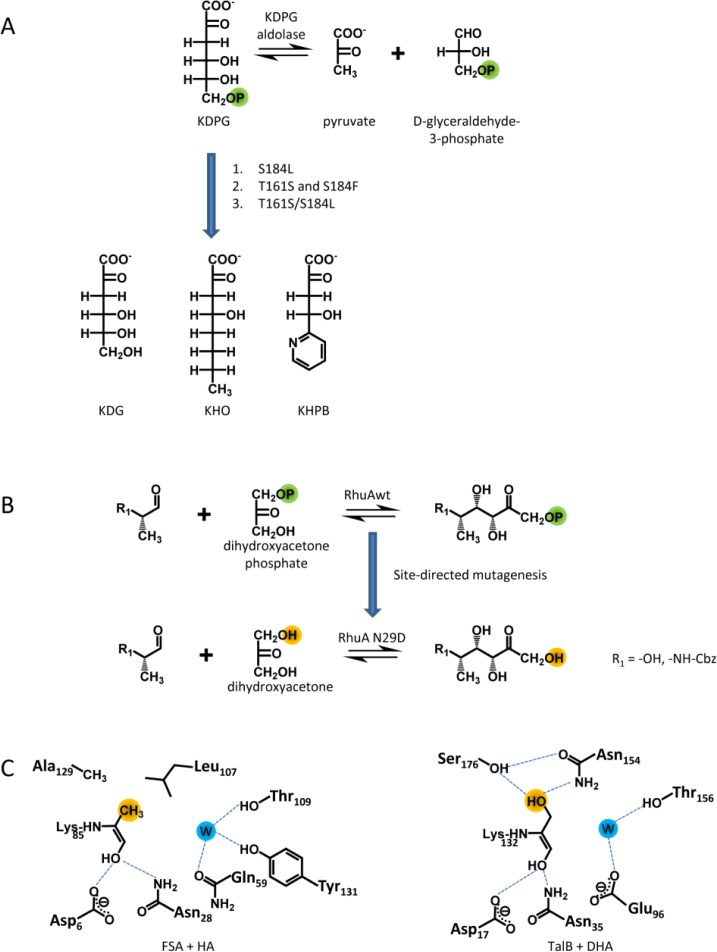
**Alteration of substrate specificity**. Protein engineering towards an increased affinity for non-phosphorylated acceptor and donor substrates in KDPG aldolase (A) and RhuA (B), respectively. C, donor binding sites of FSA and TalB.

Using a similar approach, the affinity of the pyruvate dependent 2-keto-3-deoxy-6-phosphogluconate (KDPG) aldolase was increased towards non-phosphorylated acceptor substrates [16]. The KDPG aldolase variant S184L exhibits an increased catalytic efficiency (2.5 – 6.5-fold) for uncharged hydrophobic substrates without an alteration on its stereoselectivity (Fig. 2A). In a recent study, four residues in the aldehyde (acceptor) binding site (G162, G163, S184 and T161) were randomised by saturation mutagenesis and positive clones were selected using a pyruvate auxotrophic strain [17]. G162, G163 and S184 form the phosphoryl group binding site and T161 bridges the pyruvate and the aldehyde binding site. Single substitutions (T161S, S184F) lead to an improved catalytic efficiency (4 – 12-fold) for the hydrophobic substrates 2-keto-4-hydroxy-octonoate (KHO) and (4S)-2-keto-4-hydroxy-4-(2'-pyridyl)butyrate (S-KHPB) compared to wild type (wt). This improvement was even more pronounced upon a combination of the substitutions (T161S/S184L; for S-KHPB of 450-fold). Interestingly, the double mutant retained its stereoselectivity compared to wt. The hydroxyl group of residue T161 seems to be crucial for the wt stereoselectivity. Modelling of the C4-epimeric substrates KDPG and KDPGal as Schiff base intermediate into the structure of the *E. coli* enzyme, respectively, suggests that a hydrogen bond network and the correct positioning of a water molecule in the active site are important for a stereospecific proton transfer and hence, the stereoselectivity of the enzyme.

In the DHAP dependent L-rhamnulose-1-phosphate aldolase (RhuA), the five residues (N29, N32, S75, T115 and S116) forming the binding site of the phosphoryl group of the donor substrate were substituted by Asp to enable new polar contacts which might increase the affinity towards DHA [92]. The individual variants were characterised. The introduced mutation (N29D) had only a minor effect (2-fold increase) on the yield for aldol adduct formation with an non-natural acceptor which is due to a 3-fold higher V_max_ of the N29D variant compared to wt. This increase in activity might be caused by a direct interaction of the introduced Asp side chain with the C1-OH group of the donor. In summary, an aldolase was engineered that can use the inexpensive donor DHA and exhibits a complementary stereoselectivity (3*R*,4*S*) to the DHA dependent aldolases known so far, FSA and TalB F178Y (3*S*,4*R*) (Fig. 2B).

Recently, the substrate scope of FSA and TalB F178Y has been investigated with respect to the synthesis of deoxysugars [93]. This study revealed a complementary donor specificity of the two enzymes. FSA prefers hydrophobic donors, such as hydroxyacetone (HA) and 1-hydroxy-2-butanone (HB), whereas TalB F178Y strongly prefers DHA (Fig. 2C). This was rationalised by differences in sequence and polarity of the donor binding site. By a replacement of A129S in FSA, which resembles the TalB active site, the catalytic efficiency towards DHA was greatly improved (17-fold) [94]. The reciprocal substitution in TalB F178Y (TalB F178Y/S176A) resulted in an increased activity for HA [93].

**Figure 3:**
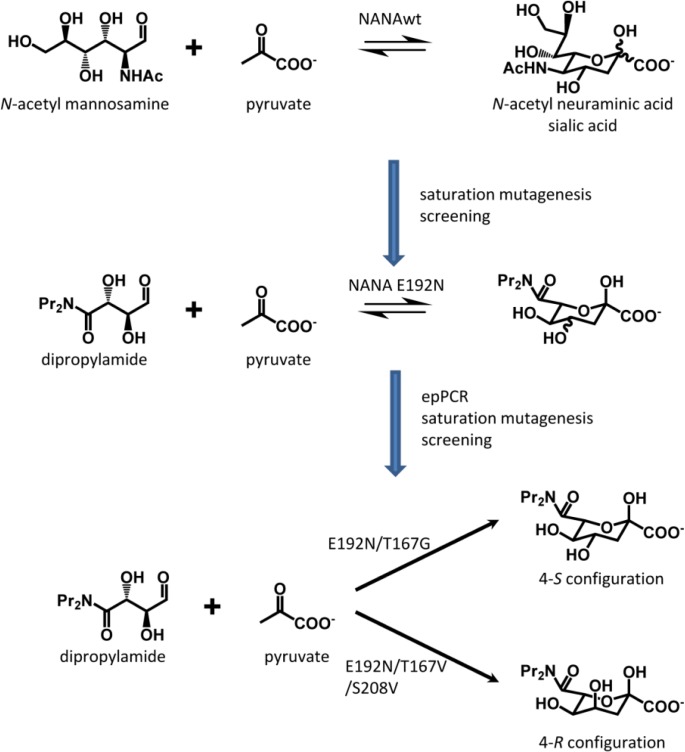
**Protein engineering of NANA**. First, NANA was engineered to use the non-natural substrate dipropylamide as acceptor. In a second approach, two stereocomplementary variants were developed.

*N*-acetylneuraminic acid aldolase (NANA) catalyses the aldol addition of pyruvate to *N*-acetylmannosamine and accepts a wide range of C5- and C6-aldehydes as substrates. It is used for the synthesis of sialic acid derivatives. To extend the substrate scope of NANA of *E. coli* a semirational approach was used [95, 96]. As there was no structure available for the *E. coli* enzyme in complex with a substrate analog the structure of a complex of a related enzyme (35% identity) was used to identify residues that interact with the acceptor substrate. At three positions (D191, E192 and S208) a saturation mutagenesis was performed and the generated libraries were screened in retro-aldol direction for pyruvate formation using a dipropylamide as model substrate (Fig. 3). The new aldolase (E192N) shows a 49-fold increase in catalytic efficiency towards the screening substrate compared to wt [96] and an almost 6-fold higher catalytic efficiency towards the new substrate than NANAwt towards its natural substrate. NANA E192N was successfully applied for the synthesis of sialic acid mimetics from substrates with differently substituted tertiary amides [95]. The products were obtained in a ≈ 80:20 mixture of the epimers.

### 3.2 Stereoselectivity

Concomitant with the C-C bond formation one or two new stereocenters are formed in an aldolase catalysed reaction. Most aldolases are strictly stereoselective but for some the stereoselectivity needs to be improved or the design of stereocomplementary enzymes is desired. For aldolases, this means that the stereochemical course of the reaction needs to be altered, i.e. the nucleophilic attack on the carbonyl carbon of the acceptor aldehyde takes place from the opposite side. Often the molecular determinants for stereoselectivity are not that well understood. Therefore, for developing a pair of stereocomplementary NANA variants [97] epPCR was applied in the first round to identify positions important for control of the stereoselectivity. As starting point the NANA E192N variant was selected which exhibits poor stereoselectivity. In the next rounds, a structure-guided approach was used. Only three (A10, T48, S208) of the residues identified by epPCR make direct contact with the substrate and were selected for separate saturation mutagenesis. Additionally, in a related aldolase (KDG aldolase) T167 forms an H-bond to the epimeric C4-OH group of the substrate and was therefore included. It turned out that the side chain at this position is very crucial for stereoselectivity. By this approach an *S*-selective (E192N/T167G) and an *R*-selective enzyme (E192N/T167V/S208V) was designed (Fig. 3). Both enzymes are about 50 times more selective (>98 : <2) than the parental enzyme.

**Figure 4:**
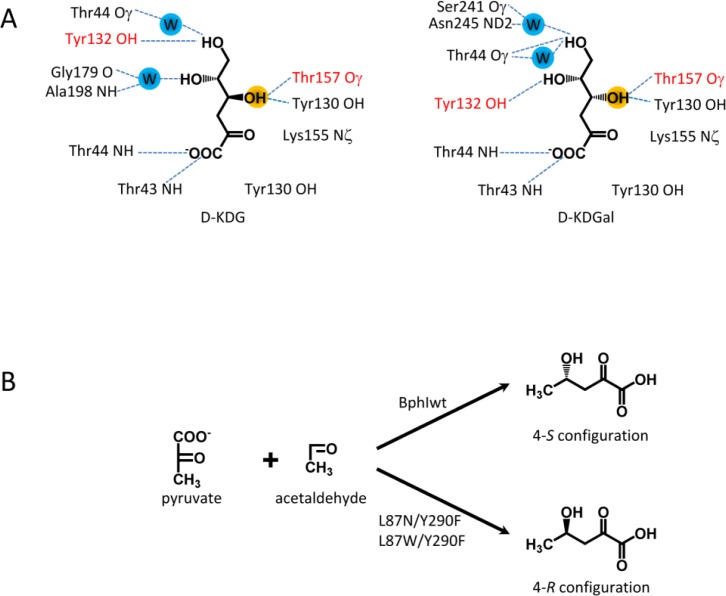
**Complementary stereoselectivity**. A, different hydrogen bond network of diastereomeric products KDGlu and KDGal in the active site of KDG aldolase from *Sulfolobus solfataricus*; B, engineering of an R-selective class II aldolase based on BphI.

The D-2-keto-3-deoxygluconate (KDG) aldolase from *Sulfolobus solfataricus* exhibits poor diastereocontrol and generates a 55:45 mixture of D-KDGlu and D-KDGal using pyruvate and D-glyceraldehyde as substrate. To improve the stereoselectivity of the enzyme and to create a pair of stereocomplementary variants X-ray structures of the aldolase with the diastereomeric products bound were employed (Fig. 4A)[15]. Interestingly, the (R)-C4-OH and (S)-C4-OH groups form similar H-bonds (T157, Y130) but the H-bond pattern for the C5-OH and C6-OH differs. A combination of saturation mutagenesis at T157 and site-directed mutagenesis was used to generate variants specific for D-KDGlu (T157C/Y132V dr 91%, T157F/Y132V dr 93%) and D-KDGal (T157V/A198L/D181Q dr 88%). This higher stereoselectivity had to be traded in by a lower affinity to the substrates (1.5 – 9 times higher K_m_) and a lower catalytic activity (60 – 100-fold drop).

The class II aldolase BphI of *Burkholderia xenovornas* is strictly stereoselective for the 4*S* isomer as most stereoselective pyruvate dependent aldolases. The aim was to design an R-selective class II aldolase. As no structure of BphI is available the structure of an ortholog (DmpG) was used and the substrate 4-hydroxy-2-oxopentanoate was modeled into the active site of DmpG [71]. According to the model, residues L87 and Y290 of BphI should be in vicinity to C4. These residues were targeted by site-directed mutagenesis (Fig. 4B). The double mutants (L87N/Y290F and L87W/Y290F) were selective for the *R*-isomer but at the cost of lower activity and affinity (effect on k_cat_/K_m_ ≤ 10-fold in aldol addition reactions compared to wt).

### 3.3 Change in reaction type

FSA, although an aldolase, belongs to the enzyme family of transaldolases. Therefore, the question is what makes this enzyme an aldolase and not a transaldolase. A structure-guided sequence alignment of FSA and TalB was used to identify positions close to the active site that differ between those two enzymes [46]. These positions were targeted by saturation mutagenesis in TalB and the generated mutant libraries were screened for formation of fructose-6-phosphate from DHA and glyceraldehyde-3-phosphate. For the aldol addition reaction, the isolated variant TalBF178Y shows a 70-fold improvement in activity compared to TalBwt and a similar catalytic efficiency as FSAwt (Fig. 1). Hence, with just one amino acid replacement a switch in enzyme class was realised. The engineering of a DHA dependent aldolase (TalB F178Y) on the TalB scaffold is a good example how a (semi)rational approach was used to change the reaction type and even more the enzyme class.

**Figure 5:**
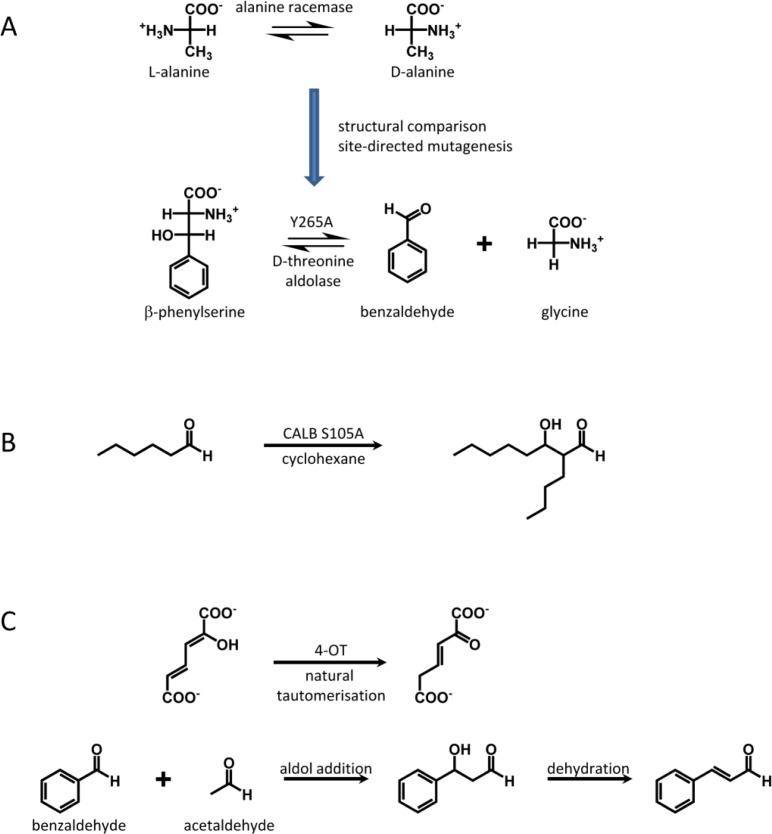
**Non-natural aldolases**. Examples for aldolase reactions catalyzed by other enzymes: A, alanine racemase, B, lipase and C, tautomerase.

By a single amino acid substitution (Y265A) the pyridoxal phosphate (PLP) dependent alanine racemase from *Geobacillus stearothermophilus* was converted into a D-threonine aldolase (Fig. 5A)[98]. Both enzymes share a common reaction intermediate (aldimine between PLP and the respective substrate). The D-threonine aldolase uses a His to abstract a proton from the cofactor bound substrate and initiate the C-C bond cleavage step. Using structural comparison of the alanine racemase from *Geobacillus stearothermophilus* and the threonine aldolase from *Thermotoga maritima* a His (H166) on the opposite side of the cofactor was identified that does not interact with the substrate directly but forms an H-bond to Y265. It was proposed that a Y265A substitution would generate more space in the active site and put H166 in the right position to act as general base in an aldol reaction. The new aldolase shows a 2.3*10^5^-fold increase in aldolase activity and a 4*10^3^-fold decrease in racemase activity with high stereoselectivity for the D-isomer.

The promiscuous activity of *Candida antarctica* lipase B (CALB, EC 3.1.1.3) for an aldol addition was enhanced by site-directed mutagenesis based on quantum chemical calculations [89]. The proposed mechanism differs from natural aldolases as the enolate intermediate is supposed to be stabilized by the oxyanion hole. By replacement of Ser105 of the catalytic triade by Ala the aldolase activity was increased 4-fold (Fig. 5B). However, the activity is much lower than of natural aldolases. But the high stability of CALB, e.g. for organic solvents, could be of advantage for biocatalytical applications.

The 4-oxalocrotonate tautomerase of *Pseudomonas putida* mt-2 exhibits a promiscuous aldolase and dehydratase activity for the formation of cinnamaldehyde from acetaldehyde and benzaldehyde (Fig. 5C)[99]. Here, the N-terminal Pro acts as nucleophile and forms an enamine intermediate with acetaldehyde. The catalytic activity was improved 16-fold (L8R [99]) and 600-fold (F50A k_cat_/K_m_ 0.5 M^-1^ s^-1^[100]) by a single point mutation.

### 3.4. De novo design

**Figure 6:**
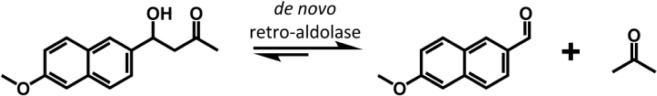
**Screening reaction used for** ***de novo*** **design of a retro-aldolase**. The retro-aldol activity was monitored in 96-well plates by cleavage of a non-natural substrate. Upon cleavage, a fluorogenic naphthyl derivative is released.

A retro-aldolase for a non-natural substrate was developed by *de novo* design using the RosettaMatch algorithm [90]. *De novo* design of an aldolase is especially challenging as the reaction mechanism involves multiple steps of protonation and deprotonation and a network of long charged side chains and hydrogen bonds. As starting point, the catalytic mechanism involving a Lys and a Schiff base intermediate was chosen. Four different motifs were selected varying in their interactions to stabilize a composite transition state which is simultaneously compatible to multiple transition states and reaction intermediates. 42 of the 72 experimentally tested designs exhibit retro-aldolase activity in the screening reaction (Fig. 6). The active designs occur in five different protein scaffolds. The most active design shows a 2*10^4^ enhancement over the uncatalysed reaction (k_cat_/k_uncat_) but is far less active than natural enzymes. The k_cat_ values for the active designs were around 10^-3^ min^-1^ which is at the lower end of the range of k_cat_ values for the catalytic antibody 38C2 (10^-3^ – 5 min^-1^) [101]. In contrast, natural aldolases exhibit k_cat_ values around 10^3^ min^-1^ [102]. The most active designs include a water molecule in the active site which is coordinated by a Tyr residue and mediates the protonation and deprotonation steps. A similar scenario is found in native aldolases, e.g. FSA. However, a later study [103] revealed that the coordination of this water molecule by a Tyr residue in the active site does not contribute to the rate enhancement. A replacement of the Tyr by Phe resulted even in an increased activity. The lowering of the pK_a_ value of the catalytic lysine residue by the surrounding hydrophobic pocket seems to have an effect on the rate enhancement and the largest contribution stems from the interaction of the substrate with its hydrophobic binding pocket [103].

Molecular dynamic simulations highlighted the importance to include protein dynamics and fluctuation as well as the orientation of the substrate in the active site in early stages of *de novo* design approaches [86]. Therefore, in a recent study [85] the design process was repeated for the motif comprising a Lys in a hydrophobic pocket and a water molecule but more care was taken for the rotamer sampling, the preorganization and positioning of side chains and packing of the active site. This resulted in a reproducible design of retro-aldolases with a very high success rate of 75%, i.e. 75% of the experimentally tested designs exhibited rates >10-fold compared to the uncatalysed reaction in buffer. But still the designed retro-aldolases are not more active than the ones in the original study [90]. So the question retained how can the gap in activity be closed between designed and natural enzymes. Optimisation of the designed aldolases by several rounds of mutagenesis and screening resulted in <100-fold increase in k_cat_/K_m_ (12 M^-1^ s^-1^) [85, 104]. These investigations revealed as limiting factor: (i) low specificity and hence inhibition by products, (ii) hydrophobic packing and positioning of substrate, (iii) hydrophobic packing and positioning of catalytic Lys which affects its reactivity.

## Outlook and conclusion

The integration of sequence and structure information for the generation of focused libraries was widely applied for the protein engineering of aldolases. However, for some enzymatic properties such as the stereoselectivity the molecular determinants are still not well understood. The synthesis of enantiopure products is one big advantage of the application of enzymes compared to “classical” organic chemistry. But not all enzymes are strictly stereoselective, especially not with non-natural substrates, and not for all possible stereo configurations a corresponding enzyme exists (e.g. DHA dependent aldolases). Future protein engineering studies will try to generate new aldolases and give more insights on the molecular determinants for stereoselectivity.

Systematic analysis of sequence was not much exploited for aldolases and transaldolases. We are currently setting up a database for the transaldolase family to get more information about subfamily specific residues, and the natural diversity of aldolases. This might allow us to discover new aldolases with interesting properties for biocatalytic applications.

Although *de novo* design of retro-aldolases gave promising results the catalytic activity even of the optimised variants is still orders of magnitude lower than of natural aldolases. Therefore, the computational tools need to be improved to close the gap in activity between the designed and native enzymes. It is not clear whether protein engineering or evolution can close this gap or if we need a better design as starting point. Especially, reactions involving multiple steps such as the aldolase reaction are challenging. Here, each step needs to be considered and not only the rate-limiting step for the natural enzyme. Long charged side chains need to be positioned correctly and a water and H-bond network needs to be designed. Considering the molecular dynamics is important as proteins are not rigid scaffolds. Furthermore, the specificity of the designed aldolases needs to be improved as product inhibition was a problem.

## References

[1] Clapes, P, Fessner, WD, Sprenger, GA, Samland, AK. (2010) Recent progress in stereoselective synthesis with aldolases. Curr Opin Chem Biol 14: 154–167 20071212 10.1016/j.cbpa.2009.11.029

[2] Clapes, P, Garrabou, X. (2011) Current trends in asymmetric synthesis with aldolases. Adv Synth Catal 353: 2263–2283

[3] Dean, SM, Greenberg, WA, Wong, CH. (2007) Recent advances in aldolase-catalyzed asymmetric synthesis. Adv Synth Catal 349: 1308–1320

[4] Samland, AK, Sprenger, GA. (2006) Microbial aldolases as C-C bonding enzymes-unknown treasures and new developments. Appl Microbiol Biotechnol 71: 253–264 16614860 10.1007/s00253-006-0422-6

[5] Jia, J, Huang, W, Schörken, U, Sahm, H, Sprenger, GA, et al. (1996) Crystal structure of transaldolase B from *Escherichia coli* suggests a circular permutation of the alpha/beta barrel within the class I aldolase family. Structure 4: 715–724 8805555 10.1016/s0969-2126(96)00077-9

[6] Samland, AK, Sprenger, GA. (2009) Transaldolase: from biochemistry to human disease. Int J Biochem Cell Biol 41: 1482–94 19401148 10.1016/j.biocel.2009.02.001

[7] Samland, AK, Rale, M, Sprenger, GA, Fessner, WD. (2011) The transaldolase family: new synthetic opportunities from an ancient enzyme scaffold. ChemBioChem 12: 1454–74 21574238 10.1002/cbic.201100072

[8] Otten, LG, Hollmann, F, Arends, IW. (2009) Enzyme engineering for enantioselectivity: from trial-and-error to rational design?. Trends Biotechnol 28: 46–54 19913316 10.1016/j.tibtech.2009.10.001

[9] Luetz, S, Giver, L, Lalonde, J. (2008) Engineered enzymes for chemical production. Biotechnol Bioeng 101: 647–53 18814289 10.1002/bit.22077

[10] Zhang, K, Sawaya, MR, Eisenberg, DS, Liao, JC. (2008) Expanding metabolism for biosynthesis of nonnatural alcohols. Proc Natl Acad Sci U S A 105: 20653–8 19064911 10.1073/pnas.0807157106 PMC2634914

[11] Bolt, A, Berry, A, Nelson, A. (2008) Directed evolution of aldolases for exploitation in synthetic organic chemistry. Arch Biochem Biophys 474: 318–30 18230325 10.1016/j.abb.2008.01.005 PMC2431125

[12] Lutz, S. (2010) Beyond directed evolution--semi-rational protein engineering and design. Curr Opin Biotechnol 21: 734–43 20869867 10.1016/j.copbio.2010.08.011 PMC2982887

[13] Reetz, MT, Wilensek, S, Zha, D, Jaeger, KE. (2001) Directed Evolution of an Enantioselective Enzyme through Combinatorial Multiple-Cassette Mutagenesis. Angew Chem Int Ed Engl 40: 3589–3591 11592190 10.1002/1521-3773(20011001)40:19<3589::AID-ANIE3589>3.0.CO;2-X

[14] Seifert, A, Pleiss, J. (2009) Identification of selectivity-determining residues in cytochrome P450 monooxygenases: a systematic analysis of the substrate recognition site 5. Proteins 74: 1028–35 18814300 10.1002/prot.22242

[15] Royer, SF, Haslett, L, Crennell, SJ, Hough, DW, Danson, MJ, et al. (2010) Structurally informed site-directed mutagenesis of a stereochemically promiscuous aldolase to afford stereochemically complementary biocatalysts. J Am Chem Soc 132: 11753–8 20684556 10.1021/ja104412a

[16] Cheriyan, M, Toone, EJ, Fierke, CA. (2007) Mutagenesis of the phosphate-binding pocket of KDPG aldolase enhances selectivity for hydrophobic substrates. Protein Sci 16: 2368–77 17962400 10.1110/ps.073042907 PMC2211689

[17] Cheriyan, M, Toone, EJ, Fierke, CA. (2012) Improving upon nature: active site remodeling produces highly efficient aldolase activity toward hydrophobic electrophilic substrates. Biochemistry 51: 1658–68 22316217 10.1021/bi201899b PMC3315183

[18] Schneider, S, Gutierrez, M, Sandalova, T, Schneider, G, Clapes, P, et al. (2010) Redesigning the Active Site of Transaldolase TalB from *Escherichia coli*: New Variants with Improved Affinity towards Nonphosphorylated Substrates. Chembiochem 11: 681–690 20148428 10.1002/cbic.200900720

[19] Li, QS, Schwaneberg, U, Fischer, M, Schmitt, J, Pleiss, J, et al. (2001) Rational evolution of a medium chain-specific cytochrome P-450 BM-3 variant. Biochim Biophys Acta 1545: 114–21 11342037 10.1016/s0167-4838(00)00268-5

[20] Reetz, MT, Kahakeaw, D, Lohmer, R. (2008) Addressing the numbers problem in directed evolution. Chembiochem 9: 1797–804 18567049 10.1002/cbic.200800298

[21] Barak, Y, Nov, Y, Ackerley, DF, Matin, A. (2008) Enzyme improvement in the absence of structural knowledge: a novel statistical approach. Isme J 2: 171–9 18253133 10.1038/ismej.2007.100

[22] Chaparro-Riggers, JF, Polizzi, KM, Bommarius, AS. (2007) Better library design: data-driven protein engineering. Biotechnol J 2: 180–91 17183506 10.1002/biot.200600170

[23] Fox, RJ, Davis, SC, Mundorff, EC, Newman, LM, Gavrilovic, V, et al. (2007) Improving catalytic function by ProSAR-driven enzyme evolution. Nat Biotech 25: 338–344 10.1038/nbt1286 17322872

[24] Pavelka, A, Chovancova, E, Damborsky, J. (2009) HotSpot Wizard: a web server for identification of hot spots in protein engineering. Nucleic Acids Res 37: W376–83 19465397 10.1093/nar/gkp410 PMC2703904

[25] Porter, CT, Bartlett, GJ, Thornton, JM. (2004) The Catalytic Site Atlas: a resource of catalytic sites and residues identified in enzymes using structural data. Nucleic Acids Res 32: D129–33 14681376 10.1093/nar/gkh028 PMC308762

[26] Dundas, J, Ouyang, Z, Tseng, J, Binkowski, A, Turpaz, Y, et al. (2006) CASTp: computed atlas of surface topography of proteins with structural and topographical mapping of functionally annotated residues. Nucleic Acids Res 34: W116–8 16844972 10.1093/nar/gkl282 PMC1538779

[27] Petrek, M, Otyepka, M, Banas, P, Kosinova, P, Koca, J, et al. (2006) CAVER: a new tool to explore routes from protein clefts, pockets and cavities. BMC Bioinformatics 7: 316 16792811 10.1186/1471-2105-7-316 PMC1539030

[28] Altschul, SF, Gish, W, Miller, W, Myers, EW, Lipman, DJ. (1990) Basic Local Alignment Search Tool. J of Mol Biol 215: 403–410 2231712 10.1016/S0022-2836(05)80360-2

[29] Edgar, RC. (2004) MUSCLE: a multiple sequence alignment method with reduced time and space complexity. BMC Bioinformatics 5: 113 15318951 10.1186/1471-2105-5-113 PMC517706

[30] Apweiler, R, Bairoch, A, Wu, CH, Barker, WC, Boeckmann, B, et al. (2004) UniProt: the Universal Protein knowledgebase. Nucleic Acids Res 32: D115–9 14681372 10.1093/nar/gkh131 PMC308865

[31] Sayers, EW, Barrett, T, Benson, DA, Bryant, SH, Canese, K, et al. (2009) Database resources of the National Center for Biotechnology Information. Nucleic Acids Res 37: D5–15 18940862 10.1093/nar/gkn741 PMC2686545

[32] Berman, HM, Westbrook, J, Feng, Z, Gilliland, G, Bhat, TN, et al. (2000) The Protein Data Bank. Nucleic Acids Res 28: 235–242 10592235 10.1093/nar/28.1.235 PMC102472

[33] Carroll SM, Ortlund EA, Thornton JW Mechanisms for the evolution of a derived function in the ancestral glucocorticoid receptor. PLoS Genet 7: e1002117 21698144 10.1371/journal.pgen.1002117 PMC3116920

[34] Steipe, B, Schiller, B, Pluckthun, A, Steinbacher, S. (1994) Sequence Statistics Reliably Predict Stabilizing Mutations in a Protein Domain. J Mol Biol 240: 188–192 8028003 10.1006/jmbi.1994.1434

[35] Lehmann, M, Pasamontes, L, Lassen, SF, Wyss, M. (2000) The consensus concept for thermostability engineering of proteins. Biochim Biophys Acta 1543: 408–415 11150616 10.1016/s0167-4838(00)00238-7

[36] Vazquez-Figueroa, E, Yeh, V, Broering, JM, Chaparro-Riggers, JF, Bommarius, AS. (2008) Thermostable variants constructed via the structure-guided consensus method also show increased stability in salts solutions and homogeneous aqueous-organic media. Protein Eng Des Sel 21: 673–680 18799474 10.1093/protein/gzn048

[37] Dai, M, Fisher, HE, Temirov, J, Kiss, C, Phipps, ME, et al. (2007) The creation of a novel fluorescent protein by guided consensus engineering. Protein Eng Des Sel 20: 69–79 17277006 10.1093/protein/gzl056

[38] Bershtein, S, Tawfik, DS. (2008) Advances in laboratory evolution of enzymes. Curr Opin Chem Biol 12: 151–8 18284924 10.1016/j.cbpa.2008.01.027

[39] Fodor, AA, Aldrich, RW. (2004) Influence of conservation on calculations of amino acid covariance in multiple sequence alignments. Proteins 56: 211–21 15211506 10.1002/prot.20098

[40] Halperin, I, Wolfson, H, Nussinov, R. (2006) Correlated mutations: advances and limitations. A study on fusion proteins and on the Cohesin-Dockerin families. Proteins 63: 832–45 16508975 10.1002/prot.20933

[41] Kuipers, RKP, Joosten, HJ, Verwiel, E, Paans, S, Akerboom, J, et al. (2009) Correlated mutation analyses on super-family alignments reveal functionally important residues. Proteins 76: 608–616 19274741 10.1002/prot.22374

[42] Tartaglia, GG, Pawar, AP, Campioni, S, Dobson, CM, Chiti, F, et al. (2008) Prediction of aggregation-prone regions in structured proteins. J Mol Biol 380: 425–36 18514226 10.1016/j.jmb.2008.05.013

[43] Buell, AK, Tartaglia, GG, Birkett, NR, Waudby, CA, Vendruscolo, M, et al. (2009) Position-dependent electrostatic protection against protein aggregation. Chembiochem 10: 1309–12 19415709 10.1002/cbic.200900144

[44] Pezza, JA, Choi, KH, Berardini, TZ, Beernink, PT, Allen, KN, et al. (2003) Spatial clustering of isozyme-specific residues reveals unlikely determinants of isozyme specificity in fructose-1,6-bisphosphate aldolase. J Biol Chem 278: 17307–13 12611890 10.1074/jbc.M209185200

[45] Barbosa, JA, Smith, BJ, DeGori, R, Ooi, HC, Marcuccio, SM, et al. (2000) Active site modulation in the N-acetylneuraminate lyase sub-family as revealed by the structure of the inhibitor-complexed *Haemophilus influenzae* enzyme. J Mol Biol 303: 405–21 11031117 10.1006/jmbi.2000.4138

[46] Schneider, S, Sandalova, T, Schneider, G, Sprenger, GA, Samland, AK. (2008) Replacement of a Phenylalanine by a Tyrosine in the Active Site Confers Fructose-6-phosphate Aldolase Activity to the Transaldolase of *Escherichia coli* and Human Origin. J Biol Chem 283: 30064–72 18687684 10.1074/jbc.M803184200 PMC2662071

[47] Scheer, M, Grote, A, Chang, A, Schomburg, I, Munaretto, C, et al. (2011) BRENDA, the enzyme information system in 2011. Nucleic Acids Res 39: D670–D676 21062828 10.1093/nar/gkq1089 PMC3013686

[48] Fischer, M, Thai, QK, Grieb, M, Pleiss, J. (2006) DWARF--a data warehouse system for analyzing protein families. BMC Bioinformatics 7: 495 17094801 10.1186/1471-2105-7-495 PMC1647292

[49] Joosten HJ. (2007) 3DM: from data to medicine, Ph.D. thesis, Wageningen University

[50] Fischer, M, Pleiss, J. (2003) The Lipase Engineering Database: a navigation and analysis tool for protein families. Nucleic Acids Res 31: 319–21 12520012 10.1093/nar/gkg015 PMC165462

[51] Racolta, S, Juhl, PB, Sirim, D, Pleiss, J. (2012) The triterpene cyclase protein family: A systematic analysis. Proteins 80: 2009–19 22488823 10.1002/prot.24089

[52] Widmann, M, Radloff, R, Pleiss, J. (2010) The Thiamine diphosphate dependent Enzyme Engineering Database: A tool for the systematic analysis of sequence and structure relations. BMC Biochemistry 11: 9 20122171 10.1186/1471-2091-11-9 PMC2831816

[53] Widmann, M, Pleiss, J, Oelschlaeger, P. (2012) Systematic Analysis of Metallo-beta-lactamases Using an Automated Database. Antimicrob Agents Chemother 56: 3481–91 22547615 10.1128/AAC.00255-12 PMC3393435

[54] Kuipers, RK, Joosten, HJ, van Berkel, WJH, Leferink, NGH, Rooijen, E, et al. (2010) 3DM: Systematic analysis of heterogeneous superfamily data to discover protein functionalities. Proteins-Structure Function and Bioinformatics 78: 2101–2113 10.1002/prot.22725 20455266

[55] Kourist, R, Jochens, H, Bartsch, S, Kuipers, R, Padhi, SK, et al. (2010) The alpha/beta-Hydrolase Fold 3DM Database (ABHDB) as a Tool for Protein Engineering. Chembiochem 11: 1635–1643 20593436 10.1002/cbic.201000213

[56] Dolan, MA, Noah, JW, Hurt, D. (2012) Comparison of common homology modeling algorithms: application of user-defined alignments. Methods Mol Biol 857: 399–414 22323232 10.1007/978-1-61779-588-6_18

[57] Arnold, K, Bordoli, L, Kopp, J, Schwede, T. (2006) The SWISS-MODEL workspace: a web-based environment for protein structure homology modelling. Bioinformatics 22: 195–201 16301204 10.1093/bioinformatics/bti770

[58] Marti-Renom, MA, Stuart, AC, Fiser, A, Sanchez, R, Melo, F, et al. (2000) Comparative protein structure modeling of genes and genomes. Annu Rev Biophys Biomol Struct 29: 291–325 10940251 10.1146/annurev.biophys.29.1.291

[59] Rohl, CA, Strauss, CE, Chivian, D, Baker, D. (2004) Modeling structurally variable regions in homologous proteins with rosetta. Proteins 55: 656–77 15103629 10.1002/prot.10629

[60] Mariani, V, Kiefer, F, Schmidt, T, Haas, J, Schwede, T. (2011) Assessment of template based protein structure predictions in CASP9. Proteins 79 Suppl 10: 37–58 22002823 10.1002/prot.23177

[61] Perl, D, Schmid, FX. (2001) Electrostatic stabilization of a thermophilic cold shock protein. J Mol Biol 313: 343–57 11800561 10.1006/jmbi.2001.5050

[62] Schweiker, KL, Zarrine-Afsar, A, Davidson, AR, Makhatadze, GI. (2007) Computational design of the Fyn SH3 domain with increased stability through optimization of surface charge charge interactions. Protein Sci 16: 2694–702 18029422 10.1110/ps.073091607 PMC2222822

[63] Goldstein, RA. (2007) Amino-acid interactions in psychrophiles, mesophiles, thermophiles, and hyperthermophiles: insights from the quasi-chemical approximation. Protein Sci 16: 1887–95 17766385 10.1110/ps.072947007 PMC2206978

[64] Braiuca, P, Buthe, A, Ebert, C, Linda, P, Gardossi, L. (2007) Volsurf computational method applied to the prediction of stability of thermostable enzymes. Biotechnol J 2: 214–20 17203502 10.1002/biot.200600175

[65] Eijsink, VG, Bjork, A, Gaseidnes, S, Sirevag, R, Synstad, B, et al. (2004) Rational engineering of enzyme stability. J Biotechnol 113: 105–20 15380651 10.1016/j.jbiotec.2004.03.026

[66] Reetz, MT, Carballeira, JD, Vogel, A. (2006) Iterative saturation mutagenesis on the basis of B factors as a strategy for increasing protein thermostability. Angew Chem Int Ed Engl 45: 7745–51 17075931 10.1002/anie.200602795

[67] Reetz, MT, Bocola, M, Carballeira, JD, Zha, D, Vogel, A. (2005) Expanding the range of substrate acceptance of enzymes: combinatorial active-site saturation test. Angew Chem Int Ed Engl 44: 4192–6 15929154 10.1002/anie.200500767

[68] Reetz, MT, Wang, LW, Bocola, M. (2006) Directed evolution of enantioselective enzymes: iterative cycles of CASTing for probing protein-sequence space. Angew Chem Int Ed Engl 45: 1236–41 16411254 10.1002/anie.200502746

[69] Marechal, JD, Kemp, CA, Roberts, GC, Paine, MJ, Wolf, CR, et al. (2008) Insights into drug metabolism by cytochromes P450 from modelling studies of CYP2D6-drug interactions. Br J Pharmacol 153 Suppl 1: S82–9 18026129 10.1038/sj.bjp.0707570 PMC2268062

[70] Sousa, SF, Fernandes, PA, Ramos, MJ. (2006) Protein-ligand docking: current status and future challenges. Proteins 65: 15–26 16862531 10.1002/prot.21082

[71] Baker, P, Seah, SY. (2012) Rational design of stereoselectivity in the class II pyruvate aldolase BphI. J Am Chem Soc 134: 507–13 22081904 10.1021/ja208754r

[72] Kapoli, P, Axarli, IA, Platis, D, Fragoulaki, M, Paine, M, et al. (2008) Engineering sensitive glutathione transferase for the detection of xenobiotics. Biosens Bioelectron 24: 498–503 18691867 10.1016/j.bios.2008.06.037

[73] Miura, S, Ferri, S, Tsugawa, W, Kim, S, Sode, K. (2008) Development of fructosyl amine oxidase specific to fructosyl valine by site-directed mutagenesis. Protein Eng Des Sel 21: 233–9 18239075 10.1093/protein/gzm047

[74] Zhu, D, Yang, Y, Majkowicz, S, Pan, TH, Kantardjieff, K, et al. (2008) Inverting the enantioselectivity of a carbonyl reductase via substrate-enzyme docking-guided point mutation. Org Lett 10: 525–8 18205368 10.1021/ol702638j

[75] Benkovic, SJ, Hammes, GG, Hammes-Schiffer, S. (2008) Free-energy landscape of enzyme catalysis. Biochemistry 47: 3317–21 18298083 10.1021/bi800049z

[76] Oelschlaeger, P, Mayo, SL, Pleiss, J. (2005) Impact of remote mutations on metallo-beta-lactamase substrate specificity: implications for the evolution of antibiotic resistance. Protein Sci 14: 765–74 15722450 10.1110/ps.041093405 PMC2279297

[77] Pavlova, M, Klvana, M, Prokop, Z, Chaloupkova, R, Banas, P, et al. (2009) Redesigning dehalogenase access tunnels as a strategy for degrading an anthropogenic substrate. Nat Chem Biol 5: 727–33 19701186 10.1038/nchembio.205

[78] Guieysse, D, Cortes, J, Puech-Guenot, S, Barbe, S, Lafaquiere, V, et al. (2008) A structure-controlled investigation of lipase enantioselectivity by a path-planning approach. Chembiochem 9: 1308–17 18418817 10.1002/cbic.200700548

[79] Morra, G, Colombo, G. (2008) Relationship between energy distribution and fold stability: Insights from molecular dynamics simulations of native and mutant proteins. Proteins 72: 660–72 18247351 10.1002/prot.21963

[80] Gatti-Lafranconi P, Natalello A, Rehm S, Doglia SM, Pleiss J, et al. Evolution of stability in a cold-active enzyme elicits specificity relaxation and highlights substrate-related effects on temperature adaptation. J Mol Biol 395: 155–66 19850050 10.1016/j.jmb.2009.10.026

[81] Henke, E, Bornscheuer, UT, Schmid, RD, Pleiss, J. (2003) A molecular mechanism of enantiorecognition of tertiary alcohols by carboxylesterases. Chembiochem 4: 485–93 12794858 10.1002/cbic.200200518

[82] Veld, MA, Fransson, L, Palmans, AR, Meijer, EW, Hult, K. (2009) Lactone size dependent reactivity in *Candida antarctica* lipase B: a molecular dynamics and docking study. Chembiochem 10: 1330–4 19425034 10.1002/cbic.200900128

[83] Stjernschantz, E, Vermeulen, NP, Oostenbrink, C. (2008) Computational prediction of drug binding and rationalisation of selectivity towards cytochromes P450. Expert Opin Drug Metab Toxicol 4: 513–27 18484912 10.1517/17425255.4.5.513

[84] Branco, RJ, Seifert, A, Budde, M, Urlacher, VB, Ramos, MJ, et al. (2008) Anchoring effects in a wide binding pocket: the molecular basis of regioselectivity in engineered cytochrome P450 monooxygenase from *B. megaterium*. Proteins 73: 597–607 18473391 10.1002/prot.22083

[85] Althoff, EA, Wang, L, Jiang, L, Giger, L, Lassila, JK, et al. (2012) Robust design and optimization of retroaldol enzymes. Prot Sci 21: 717–726 10.1002/pro.2059 PMC3403469 22407837

[86] Ruscio, JZ, Kohn, JE, Ball, KA, Head-Gordon, T. (2009) The Influence of Protein Dynamics on the Success of Computational Enzyme Design. J Am Chem Soc 131: 14111–14115 19788332 10.1021/ja905396s PMC2918245

[87] Bornscheuer, UT, Kazlauskas, RJ. (2004) Catalytic promiscuity in biocatalysis: using old enzymes to form new bonds and follow new pathways. Angew Chem Int Ed Engl 43: 6032–40 15523680 10.1002/anie.200460416

[88] Nobeli, I, Favia, AD, Thornton, JM. (2009) Protein promiscuity and its implications for biotechnology. Nat Biotechnol 27: 157–67 19204698 10.1038/nbt1519

[89] Branneby, C, Carlqvist, P, Magnusson, A, Hult, K, Brinck, T, et al. (2003) Carbon-carbon bonds by hydrolytic enzymes. J Am Chem Soc 125: 874–5 12537478 10.1021/ja028056b

[90] Jiang, L, Althoff, EA, Clemente, FR, Doyle, L, Rothlisberger, D, et al. (2008) De novo computational design of retro-aldol enzymes. Science 319: 1387–91 18323453 10.1126/science.1152692 PMC3431203

[91] Wolfenden, R, Snider, MJ. (2001) The depth of chemical time and the power of enzymes as catalysts. Acc Chem Res 34: 938–45 11747411 10.1021/ar000058i

[92] Garrabou, X, Joglar, J, Parella, T, Crehuet, R, Bujons, J, et al. (2011) Redesign of the Phosphate Binding Site of L-Rhamnulose-1-Phosphate Aldolase towards a Dihydroxyacetone Dependent Aldolase. Adv Synth Catal 353: 89–99

[93] Rale, M, Schneider, S, Sprenger, GA, Samland, AK, Fessner, WD. (2011) Broadening Deoxysugar Glycodiversity: Natural and Engineered Transaldolases Unlock a Complementary Substrate Space. Chem Eur J 17: 2623–2632 21290439 10.1002/chem.201002942

[94] Castillo, JA, Guerard-Helaine, C, Gutierrez, M, Garrabou, X, Sancelme, M, et al. (2010) A Mutant D-Fructose-6-Phosphate Aldolase (Ala129Ser) with Improved Affinity towards Dihydroxyacetone for the Synthesis of Polyhydroxylated Compounds. Adv Synth Catal 352: 1039–1046

[95] Woodhall, T, Williams, G, Berry, A, Nelson, A. (2005) Creation of a tailored aldolase for the parallel synthesis of sialic acid mimetics. Angew Chem Int Ed Engl 44: 2109–12 15742315 10.1002/anie.200462733

[96] Williams, GJ, Woodhall, T, Nelson, A, Berry, A. (2005) Structure-guided saturation mutagenesis of N-acetylneuraminic acid lyase for the synthesis of sialic acid mimetics. Protein Eng Des Sel 18: 239–46 15897188 10.1093/protein/gzi027

[97] Williams, GJ, Woodhall, T, Farnsworth, LM, Nelson, A, Berry, A. (2006) Creation of a pair of stereochemically complementary biocatalysts. J Am Chem Soc 128: 16238–47 17165777 10.1021/ja065233q

[98] Seebeck, FP, Hilvert, D. (2003) Conversion of a PLP-dependent racemase into an aldolase by a single active site mutation. J Am Chem Soc 125: 10158–9 12926923 10.1021/ja036707d

[99] Zandvoort, E, Baas, BJ, Quax, WJ, Poelarends, GJ. (2011) Systematic screening for catalytic promiscuity in 4-oxalocrotonate tautomerase: enamine formation and aldolase activity. Chembiochem 12: 602–9 21290551 10.1002/cbic.201000633

[100] Zandvoort, E, Geertsema, EM, Quax, WJ, Poelarends, GJ. (2012) Enhancement of the Promiscuous Aldolase and Dehydration Activities of 4-Oxalocrotonate Tautomerase by Protein Engineering. ChemBioChem 13: 1274–1277 22615135 10.1002/cbic.201200225

[101] Barbas, CF, Heine, A, Zhong, GF, Hoffmann, T, Gramatikova, S, et al. (1997) Immune versus natural selection: Antibody aldolases with enzymic rates but broader scope. Science 278: 2085–2092 9405338 10.1126/science.278.5346.2085

[102] Choi, KH, Mazurkie, AS, Morris, AJ, Utheza, D, Tolan, DR, et al. (1999) Structure of a fructose-1,6-bis(phosphate) aldolase liganded to its natural substrate in a cleavage-defective mutant at 2.3 angstrom. Biochemistry 38: 12655–12664 10504235 10.1021/bi9828371

[103] Lassila, JK, Baker, D, Herschlag, D. (2010) Origins of catalysis by computationally designed retroaldolase enzymes. Proc Natl Acad Sci U S A 107: 4937–42 20194782 10.1073/pnas.0913638107 PMC2841948

[104] Wang, L, Althoff, EA, Bolduc, J, Jiang, L, Moody, J, et al. (2012) Structural analyses of covalent enzyme-substrate analog complexes reveal strengths and limitations of de novo enzyme design. J Mol Biol 415: 615–25 22075445 10.1016/j.jmb.2011.10.043 PMC3440004

